# Travel-associated neurological disease terminated in a *postmortem* diagnosed atypical HSV-1 encephalitis after high-dose steroid therapy – a case report

**DOI:** 10.1186/s12879-020-4859-5

**Published:** 2020-02-18

**Authors:** Andreas Osterman, Viktoria C. Ruf, Cristina Domingo, Andreas Nitsche, Peter Eichhorn, Hanna Zimmermann, Klaus Seelos, Sabine Zange, Konstantinos Dimitriadis, Hans-Walter Pfister, Thorsten Thye, Armin Giese, Dennis Tappe, Stephan Böhm

**Affiliations:** 1 Max von Pettenkofer Institute, Virology, Faculty of Medicine, LMU Munich, Pettenkoferstraße 9a, D-80336 Munich, Germany; 2 German Center for Infection Research (DZIF), partner site Munich, Pettenkoferstraße 9a, D-80336 Munich, Germany; 3 Center for Neuropathology and Prion Research, Faculty of Medicine, LMU Munich, Feodor-Lynen-Straße 23, D-81377 Munich, Germany; 4 Robert Koch Institute, Center for Biological Threats and Special Pathogens, Highly Pathogenic Viruses ZBS-1, Seestraße 10, D-13353 Berlin, Germany; 5 Institute of Laboratory Medicine, University Hospital Campus Großhadern, LMU Munich, Marchioninistraße 15, D-81377 Munich, Germany; 6 Department of Neuroradiology, University Hospital Campus Großhadern, LMU Munich, Marchioninistraße 15, D-81377, Munich, Germany; 7 Bundeswehr Institute of Microbiology, Munich, Neuherbergstraße 11, D-80937 Munich, Germany; 8 Department of Neurology, University Hospital Campus Großhadern, LMU Munich, Marchioninistraße 15, D-81377 Munich, Germany; 9 Bernhard Nocht Institute for Tropical Medicine, Hamburg, Bernhard-Nocht-Straße 74, D-20359 Hamburg, Germany

**Keywords:** Herpes simplex virus, Next generation sequencing, Encephalitis, Steroid, The Gambia, Travel associated, Yellow fever vaccine associated neurological disease

## Abstract

**Background:**

Human encephalitis can originate from a variety of different aetiologies, of which infection is the most common one. The diagnostic work-up is specifically challenging in patients with travel history since a broader spectrum of unfamiliar additional infectious agents, e. g. tropical disease pathogens, needs to be considered. Here we present a case of encephalitis of unclear aetiology in a female traveller returning from Africa, who in addition developed an atypical herpes simplex virus (HSV) encephalitis in close temporal relation with high-dose steroid treatment.

**Case presentation:**

A previously healthy 48-year-old female presented with confusion syndrome and impaired vigilance which had developed during a six-day trip to The Gambia. The condition rapidly worsened to a comatose state. Extensive search for infectious agents including a variety of tropical disease pathogens was unsuccessful. As encephalitic signs persisted despite of calculated antimicrobial and antiviral therapy, high-dose corticosteroids were applied intravenously based on the working diagnosis of an autoimmune encephalitis. The treatment did, however, not improve the patient’s condition. Four days later, bihemispheric signal amplification in the insular and frontobasal cortex was observed on magnetic resonance imaging (MRI). The intracranial pressure rapidly increased and could not be controlled by conservative treatment. The patient died due to tonsillar herniation 21 days after onset of symptoms. Histological examination of *postmortem* brain tissue demonstrated a generalized lymphocytic meningoencephalitis. Immunohistochemical reactions against HSV-1/2 indicated an atypical manifestation of herpesviral encephalitis in brain tissue. Moreover, HSV-1 DNA was detected by a next-generation sequencing (NGS) metagenomics approach. Retrospective analysis of cerebrospinal fluid (CSF) and serum samples revealed HSV-1 DNA only in specimens one day *ante mortem*.

**Conclusions:**

This case shows that standard high-dose steroid therapy can contribute to or possibly even trigger fulminant cerebral HSV reactivation in a critically ill patient. Thus, even if extensive laboratory diagnostics including wide-ranging search for infectious pathogens has been performed before and remained without results, continuous re-evaluation of potential differential diagnoses especially regarding opportunistic infections or reactivation of latent infections is of utmost importance, particularly if new symptoms occur.

## Background

Clarification of the aetiology in a timely manner is crucial regarding therapy and outcome of patients presenting with symptoms of encephalitis [1], but also in terms of hygiene measures, post exposure prophylaxis of contact persons, as well as epidemiological (outbreak) control measures. The most common causes of encephalitis are infections, in which viral infections, especially herpes simplex virus type 1 (HSV-1), account for the majority of cases [2]. However, depending on the geographical region, a different spectrum of pathogens needs to be considered. The standard diagnostic procedures for patients with encephalitis include magnetic resonance imaging (MRI) or computed tomography (CT), analysis of CSF samples as well as electroencephalography (EEG). Moreover, brain biopsy may be sought to confirm an inflammatory process. NGS (next-generation sequencing) techniques are increasingly applied in diagnostics, particularly in complex and unclear cases to identify potential pathogens through metagenomic analyses. These metagenomic analyses detect any fragment of nucleic acid present in a specimen. Bioinformatic downstream analyses can then align these fragments into larger genomes and differentiate them according to human, bacterial or viral origin, for example. Apart from infections, autoantibodies in the context of autoimmune diseases or paraneoplastic syndromes have been increasingly recognized to be associated with encephalitis and have to be included into the differential diagnoses [3]. However, differentiation between infectious and autoimmune encephalitis can be challenging as there may be substantial overlap in their clinical presentation [4]. Here, we report the case of a female traveller with encephalitis of unknown aetiology after a trip to Africa who developed atypical HSV-1 encephalitis in close temporal relation with high-dose steroid therapy.

## Case presentation

**Fig. 1:**
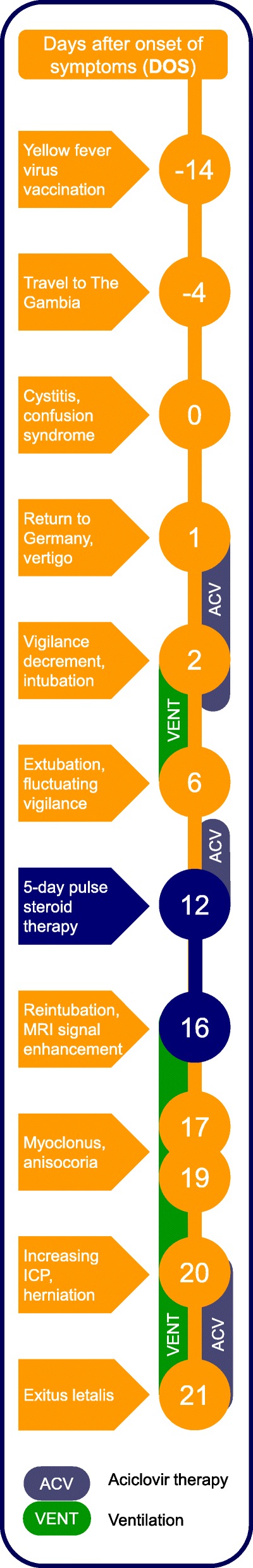
Timeline

**Fig. 2:**
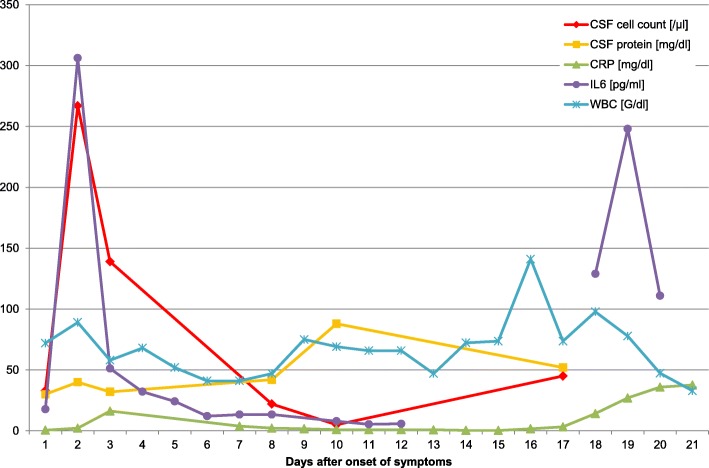
Laboratory results. Chronological visualization of relevant values from cerebrospinal fluid (cell count [< 5 cells/μl] and protein concentration [15–45 mg/dl]), serum (C-reactive protein (CRP) [< 0.5 mg/dl] and interleukin 6 (IL6) [< 5.9 pg/ml]) and white blood cell (WBC) count [40–104 G/dl]

**Fig. 3:**
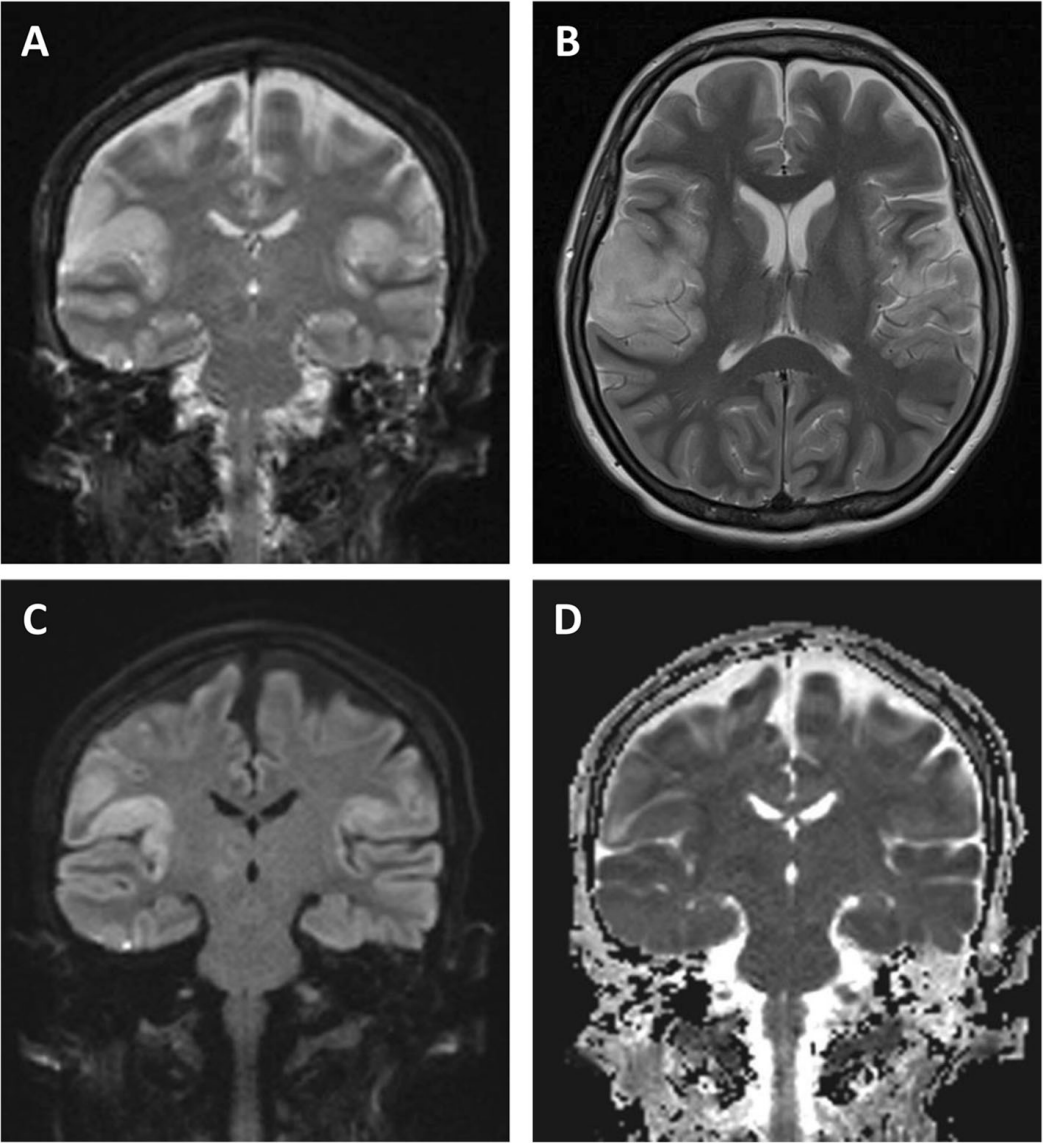
cMRI imaging. Cortical/subcortical oedema within insular and parietal lobe on coronal and axial T2w (**a, b**), coronal DWI with increased signal on B1000 image (**c**) and decreased ADC (apparent diffusion coefficient) (**d**) within the encephalitic lesion

A 48-year-old Caucasian previously healthy female without a history of recreational drug use developed confusion syndrome and haemorrhagic cystitis during a travel to The Gambia (Fig. 1). There she presented to a local hospital, where trichomoniasis was diagnosed and treated with antiparasitic chemotherapy. She decided to interrupt the trip and returned to Munich, Germany after only 6 days of travel (corresponds to “day one after onset of symptoms” (DOS 1)). Upon return, the patient was directly referred to a community hospital with dizziness, blurred vision, confusion syndrome, and upper arm pain. In the emergency department, she acutely developed a delirious state with fluctuating vigilance and had to be intubated. Laboratory testing revealed hyponatremia which was carefully corrected. While IL6 was already slightly elevated (17.7 pg/μl [< 5.9 pg/ml]), CRP (0.5 mg/dl [< 0.5 mg/dl]) and body temperature were normal, no rash or neck stiffness were observed (Fig. 2). Based on CSF pleocytosis (CSF cell count 33 cells/μl [< 5 cells/μl], CSF protein 30 mg/dl [15–45 mg/dl], CSF lactate 2.47 mmol/l [1.1–2.4 mmol/l], CSF glucose 78 mg/dl [50–90 mg/dl], intact blood-CSF barrier), empiric antibiotic and antiviral treatment with ampicillin (12 g/d i.v. for 11 days), ceftriaxone (4 g/d i.v. for 16 days) and acyclovir (750 mg i.v. tid for 3 days) was started immediately upon admission to the emergency room, however, as HSV-PCR was negative and imaging of the head and brain (CT and MRI including angiography) was unsuspicious, acyclovir treatment was ceased after 3 days. Comprehensive microbiological testing for numerous infectious pathogens including tropical and sexually transmissible pathogens, as well as search for autoantibodies were unsuccessful (see Table 1). The results of testing for yellow fever virus were compatible with a previous vaccination which had been administered 10 days before the travel to Africa. The EEG showed patterns of severe diffuse encephalopathy. Since pulmonary gas exchange and protective reflexes were adequate, the patient could be extubated and was respiratory and hemodynamically stable, though still unresponsive and alternating agitated or somnolent. Ten days after the onset of symptoms, the patient was transferred to a university hospital, where microbiological and laboratory testing were intensified. As the aetiology of the encephalitic syndrome was still unclear, steroid pulse therapy was initiated under the rationale of suspected limbic encephalitis 12 days after symptom onset and continued for 5 days, but no clinical improvement could be achieved. However, as on MRI new bihemispheric insular and frontobasal signal enhancement appeared (Fig. 3) and since in parallel inflammatory markers started increasing, empiric antibiotic therapy was again applied (meropenem 2 g i.v. tid from DOS 16 on, vancomycin i.v. [trough level 10–15 μg/dl] from DOS 18 on). Since the state of vigilance was still unchanged and protective reflexes were absent, the patient was re-intubated. Moreover, facial myoclonus emerged, which was treated with levetiracetam and phenytoin. As the anticonvulsive therapy was insufficient to control the myoclonus, therapeutic sedation was initiated, under which no spikes were detectable any more on EEG. Two days later, anisocoria (r > l) was noticed and conservative treatment of the underlying increased intracranial pressure (ICP) was started with osmotherapeutics, deep anaesthesia (including barbiturate), and hyperventilation. Neurosurgical therapy options were discussed, however, not feasible because of progressive and generalized oedematous brain swelling with transtentorial and foraminal herniation. The patient showed fixed dilated pupils and died 1 day later, 21 days after first symptom onset. As the cause of the disease was completely unclear, autoptic examination was sought which could help to explain at least the final course of the disease. Below, the most important results of the diagnostic investigations are presented and discussed in detail.

**Table 1:** Infectious disease work-up

Pathogen	DOS	Method	Specimen
Borna disease virus-1	21	PCR	CSF
Borrelia burgdorferi	10	Ig, AI	CSF, S
Chikungunya virus	10	Ig	S
Cryptococcus neoformans/gatti	10	PCR	CSF
Cytomegalovirus	2, 10	PCR, Ig, AI	CSF, S
Dengue virus	10, 11	Ig	S
Ebstein Barr virus	10	PCR, Ig, AI	CSF, S
Enterovirus	10	PCR	CSF
*Escherichia coli* K1	10	PCR	CSF
Flaviviruses	21	PCR	CSF
*Haemophilus influenzae*	10	PCR	CSF
Hepatitis A virus	10	Ig	S
Hepatitis B virus	10	Ig, Ag	S
Hepatitis C virus	10	Ig	S
Herpes simplex virus 1/2	2, 10	PCR, Ig, AI	CSF, S
Human herpes virus 6	10	PCR	S
Human immunodeficiency virus	10	Ig, Ag	S
Japanese encephalitis virus	11	Ig	S
Leptospira sp.	10	Ig	S
*Listeria monocytogenes*	10	PCR	CSF
Lymphocytic choriomeningitis virus	10	Ig	CSF, S
Measels virus	10	AI	CSF, S
*Mycobacterium tuberculosis*	17	QFT	B
Neisseria meningitidis	10	PCR	CSF
Parechovirus	10	PCR	CSF
Plasmodium sp.	19	RT, M	S
Rabies virus	10	PCR	CSF
Rift Valley virus	11	PCR, Ig	S
Rubella virus	10	AI	CSF, S
Sandfly fever virus	10	Ig	S
*Streptococcus agalactiae*	10	PCR	CSF
Streptococcus pneumonieae	10	PCR	CSF
Tick-borne encephalitis virus	10, 11	Ig	CSF, S
Treponema pallidum	2, 10	Ig	S
Trypanosoma brucei gambiense/rhodesiense	2	PCR, Ig	S
Trypanosoma cruzi	2	Ig	S
Varicella zoster virus	10	PCR, Ig, AI	CSF, S
Variegated squirrel bornavirus-1	21	PCR	CSF
West Nile virus	10, 11	Ig	S
Yellow fever virus	2, 9, 11, 17, 21	PCR, Ig	CSF, S, A
Zika virus	10	PCR, Ig	S

Evaluated pathogens with negative findings

*DOS* Days after onset of symptoms, *Ig* Serology, *AI* pathogen specific CSF/serum antibody index, *S* Serum, *Ag* Antigen-Test, *QFT* Quantiferon test, *B* Blood, *RT* Rapid test, *M* Microscopy, *A* autopsy tissue

### Diagnostic focus and assessment

#### Pathogen diagnostics

During the course of the disease, numerous laboratory examinations were performed in an attempt to identify infectious pathogens potentially responsible for the patient’s encephalitis (see Table 1). For this reason, blood, CSF, urine, stool, and respiratory samples were also repeatedly applied to microbiological cultures which did not detect any relevant pathogens. For differential diagnosis, methods were selected according to different criteria: (I) The most common pathogens were analysed by multiplex PCR from CSF (cytomegalovirus (CMV), enterovirus, herpes simplex virus-1/2 (HSV-1/2), human herpes virus 6 (HHV6), human parechovirus, varicella zoster virus (VZV), *Escherichia coli* K1, *Haemophilus influenzae*, *Listeria monocytogenes*, *Neisseria meningitidis*, *Streptococcus agalactiae*, *Streptococcus pneumoniae*, *Cryptococcus neoformans/gattii*). (II) A group of pathogens was investigated because of the travel history (chikungunya virus, dengue virus, *Mycobacterium tuberculosis*, *Plasmodium* sp., rabies virus, Rift Valley virus, sandfly fever virus, *Trypanosoma* sp., West Nile virus, yellow fever virus, Zika virus) or (III) because of a potential risk profile that could not be specifically asked (HIV, hepatitis viruses, *Treponema pallidum*). (IV) Moreover, (zoonotic) pathogens which may have been acquired before the journey (Borna disease virus 1 (BoDV-1), *Borrelia burgdorferi*, Japanese encephalitis virus, *Leptospira* sp., tick-borne encephalitis virus) or (V) which are rare or very rare pathogens of encephalitis (Ebstein Barr virus, lymphocytic choriomeningitis virus, measels virus, rubella virus) were examined.

#### Yellow fever vaccine-associated neurological disease (YEL-AND) and autoimmune encephalitis

Since the patient had received yellow fever vaccination in preparation for her travel, the presence of a yellow fever vaccine associated neurotropic syndrome was considered. Anti-yellow fever antibodies in the patient’s serum were within the range of a normal post-vaccination immune response and were not detected in the cerebrospinal fluid. No yellow fever vaccine virus RNA could be amplified from any sample (brain biopsies, cerebrospinal fluid, serum, urine). Therefore, YEL-AND could not be confirmed.

The investigation of a broad spectrum of neuronal autoantibodies (against AMPAR1/2, amphiphysin, aquaporin 4, Ca-channel, CASPR2, CV2 (CRMP5), GABARB1/2, GAD, Hu, LGI-1, Ma1/2, NMDAR, Purkinje cells, Ri, Yo) remained without pathological findings and thus, no evidence for an autoimmune-mediated encephalitis existed.

#### Autopsy

**Fig. 4:**
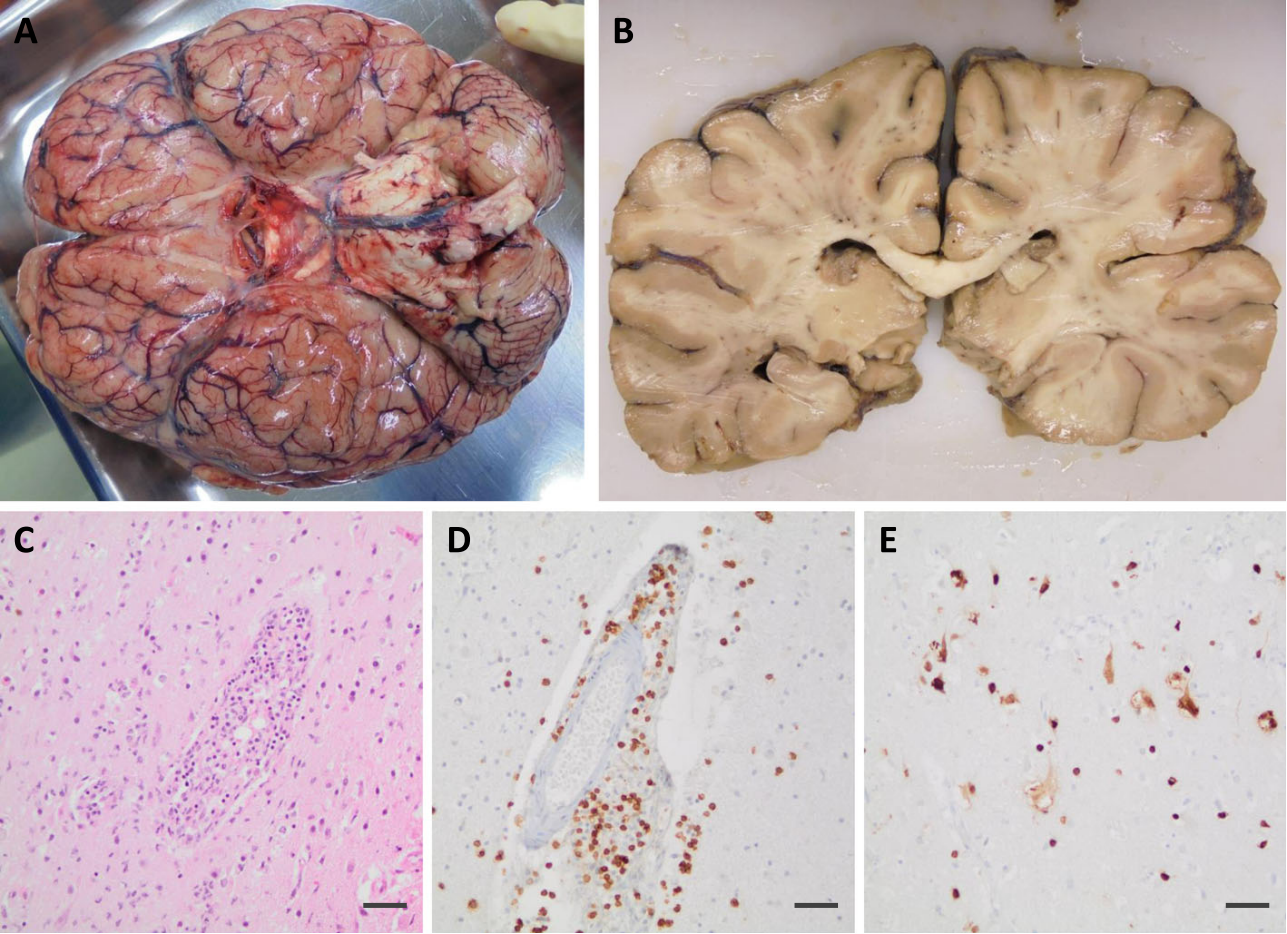
Neuropathological autopsy findings. Oedematous swelling with sulcal effacement and signs of transtentorial and tonsillar herniation (**a**). Symmetrical, though remarkably compressed ventricular system reflecting the increased intracranial pressure (**b**). Histological examination showed lymphocytic meningoencephalitis with marked perivascular and intraparenchymal lymphocyte infiltration (**c**). The infiltrates are primarily composed of CD3-positive T-lymphocytes (**d**). Numerous infected neurons demonstrated by HSV-1 immunohistochemistry (**e**). Magnification (**c**)-(**e**): 20x; Scale bar: 50 μm

On autopsy, neuropathological examination of the brain demonstrated macroscopically oedematous swelling and signs of increased intracranial pressure with sulcal effacement, uncus herniation, and discretely prominent cerebellar tonsils (Fig. 4a). Temporally accentuated haemorrhagic necrosis, which is typically observed in classical cases of herpesviral encephalitis, were not seen (Fig. 4b). Histological examination revealed lymphocytic meningoencephalitis with plenty of perivascular and intraparenchymal T-lymphocytes (Fig. 4c and d). The diagnosis of herpesviral encephalitis was made based on the immunohistochemical detection of numerous infected neurons that stained positive for HSV-1 (Fig. 4e).

#### NGS analysis

**Fig. 5:**
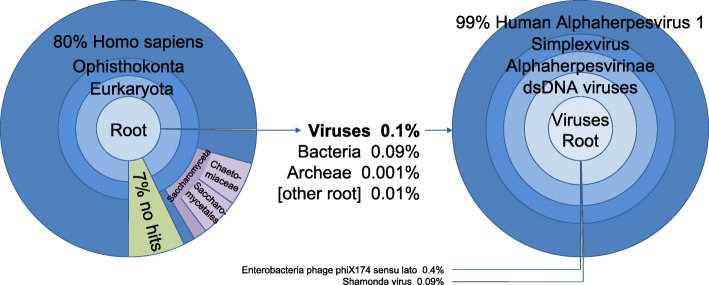
NGS analysis. RNA was transcribed in cDNA and sequencing libraries were prepared using the NexteraXT DNA preparation kit following the manufacturer’s instructions. Libraries were loaded and sequenced on an Illumina MiSeq sequencer using a V3 600 cycles kit. Taxonomic classifications of sequencing reads were performed with the Kraken software and visualized with the Krona tools. Left: Reads total, frontal lobe; Right: Reads, virus specific; frontal lobe

NGS analysis was performed with DNA and RNA isolated from native autopsy tissue of the frontal lobe and cerebellum together with preserved serum from DOS 21. The number of reads that could be assigned to viral specimens was highest in material from the frontal lobe (Table 2). The majority of those reads belonged to the human alpha-herpesvirus type 1 (HSV-1) (Fig. 5).

**Table 2:** Results of NGS analysis

Material		RNA/DNA [ng/μl]	Reads total	Reads HSV-1
Frontal lobe	RNA	0.47	4,252,804	4188 (0.10%)
Frontal lobe	DNA	1.5	2,186,683	490 (0.02%)
Cerebellum	RNA	0.39	4,983,018	0
Serum (DOS 21)	RNA	0.42	2,523,808	0

After HSV DNA and antigen were detected in autopsy specimens of the brain by NGS analysis and immunohistochemical detection, respectively, retrospective conventional single PCR analyses from archived CSF, serum and tissue were performed to confirm these results (see below and Table 3).

**Table 3:** Results of testing for HSV

DOS	Specimen	HSV Analyte	Value
2	CSF	DNA	negative
10	CSF, serum	DNA	negative
10	CSF, serum	AI	1.4
10	serum	IgG	32 AE/ml
21	CSF	DNA	1.4 Mio Geq/ml
21	serum	DNA	1000 Geq/ml
21	brain tissue	DNA	positive
21	CSF, serum	AI	0.5
21	serum	IgG	27 AE/ml

Retrospective analysis of CSF and serum samples provided HSV DNA positive PCR results only for the shorty before death collected specimens (technical limit of detection (LOD) = 700 Geq/ml, at 95% hit rate). AI = serum CSF antibody specificity index (normal range value 0.5–1.5)

## Discussion and conclusions

Patients with encephalitis need to be diagnosed and appropriately treated in a timely manner. Viral infections account for the majority of cases (42%) followed by autoimmune-mediated encephalitis (21%), while about 40% of the patients remain without a final diagnosis [2].

In our case, the clinical suspicion of encephalitis quickly emerged and was supported by results of lumbar puncture and EEG. In the initial phase of the disease, diagnostic imaging (CT and MRI) could not reveal a morphological correlate with these findings and the patient’s symptoms. However, a vascular or malignant cause of the disease could be excluded in this way. HSV encephalitis is the most common sporadic form of encephalitis in Western Europe. There is an international consensus to carry out a rapid diagnosis using PCR from the CSF and to administer a empiric therapy with acyclovir. Such measures reduce the lethality of HSV encephalitis from about 70 to 30% [5].

Empiric antibiotic and antiviral treatment was immediately started upon admission to hospital according to the current guidelines of the German Society for Neurology [6, 7], so that there was no delay in therapy initiation. Although HSV-PCR from CSF in the early phase of HSV encephalitis does not reach a sensitivity of 100% [8], acyclovir therapy was terminated after 3 days. This discontinuation of the empirical acyclovir therapy was justified by the lack of strong clinical suspicion of a very rare ‘PCR negative’ HSV encephalitis: Repeated cranial imaging (CT and MRI) without abnormal findings and the first clinical improvement allowing an extubation after 6 days were the evidence for this therapeutic decision. After transfer to the university hospital, a second HSV-PCR from CSF performed on day 10 – after an acyclovir break of several days – was also negative and underlined that HSV was extremely unlikely to be the causative pathogen of the patient’s encephalopathy presented at this time. Moreover, a repeatedly normal serum CSF antibody specificity index for HSV throughout the disease duration (21 days) showed that HSV encephalitis diagnosed *post mortem* must indeed have developed towards the end of the clinical course (Table 3).

A travel-associated infectious disease was considered an important differential diagnosis from the very first moment due to the presented neurological symptoms associated with a journey abroad [9]. All relevant pathogens were examined after an evaluation of the potential exposure risk [10]. However, in this context, it must also be discussed, whether the very short duration of the stay abroad equals an appropriately long incubation period for an acute infection. Therefore, emerging viruses that may have been acquired before the journey, such as zoonotic Borna viruses (BoDV-1 and VSBV-1), were also taken into account.

In addition to the targeted search for pathogens, the ‘extended’ microbiological diagnosis in such a travel-related case must include vaccines applied in a close temporal connection as well as medication already administered on site. A first important aspect of the patient’s travel-related vaccination record is the correctly administered yellow fever vaccination 2 weeks before departure. YEL-AND is a serious side effect after primary vaccination [11]. The frequency is given as approx. 0.8/100,000 doses. Typically, patients develop meningoencephalitis 14 (3–28) days after administration but recover without sequelae. This infection of the CNS by the vaccine virus itself can be detected by the detection of yellow fever vaccine RNA or yellow fever virus-specific antibodies in the cerebrospinal fluid [12]. Few data suggest that yellow fever vaccination may trigger an autoimmune response and cause symptoms such as Guillain-Barré syndrome, multiple sclerosis or acute demyelinating encephalomyelitis (ADEM) [13]. However, as described above, there was no evidence of YEL-AND in our case.

Another important point of the travel history is that haemorrhagic cystitis with proven *Trichomonas* infection was already treated with antibiotics during the stay in The Gambia. Even if the exact active substance was not traceable, a therapy with metronidazole seems to be very likely. Metronidazole-induced encephalopathies described in the literature differ in their typical clinical course from our case [14]. Nevertheless, we cannot exclude such a possible or other (unknown) drug side effect as the cause of the persistent impaired consciousness in the retrospective assessment of the case.

We had expected that the NGS analysis from the brain tissue samples would detect unanticipated or possibly even novel pathogens, which was, however, not the case. Nevertheless, reduced sensitivity remains a weakness of this method. In our case study, for example, an HSV load of 1000 Geq/ml detectable in serum by routine PCR could not generate HSV reads in the NGS investigation of the serum at the same point in time (compare Tables 2 and 3). This may be relevant because it cannot be excluded that the initial phase of the disease was caused by an unknown pathogen other than HSV and not by autoimmune disease. The viral load in brain tissue may have fallen below the lower detection limit of the NGS analysis over the course of the disease. Nevertheless, in our case we estimate the probability of not being able to detect a previously unknown pathogen after 21 days of illness in a brain biopsy via NGS as low.

Even today, it is estimated that approximately half of all encephalitis cases remain without identification of a clear cause [2, 15]. Despite advancing diagnostic options from multiplex PCR systems to NGS technology, this gap in clinical microbiology is not closed. Publications on NGS metagenomic analyses in patients with encephalitis show that known and common encephalitis pathogens but also rare, unexpected, or even novel organisms can be detected in patient samples [16–18].

The HSV-reactivation presented as an atypical manifestation of HSV-1 encephalitis without the temporally and frontally accentuated haemorrhagic necroses which are typically observed on imaging and autopsy. Such atypical manifestations of HSV-1 encephalitis can be observed in immunosuppressed or critically ill patients [19–21] and impede making the correct diagnosis. Histological examination demonstrated pronounced lymphocytic meningoencephalitis with immunohistochemical detection of HSV-1 antigens. Due to the extensive destruction of the brain tissue, any signs, allowing conclusions towards the aetiology of the initial disease could not be assessed.

In animal models, the hypothesis that naturally occurring stressful situations in the host lead to reactivation of latent herpes viruses due to increased endogenous corticosteroid release was supported early on [22]. The effects of corticosteroids on the gene expression of the cell and latent virus, which occur a few hours after injection, can lead to an altered balance between herpes virus and neuron and can lead to the death of the neuron by switching the virus into a lytic replication cycle, even with a single dose of dexamethasone.

Although it is well known that steroid therapy can cause HSV reactivation in neurons in animal models, only few cases have been reported in the literature in which HSV reactivation and encephalitis coincided with steroid therapy in humans [23]. Of note, most of the described treatment regimens consisted of a combination with other stress-inducing or immunosuppressive therapies such as irradiation or chemotherapy [24, 25]. Therefore, HSV reactivation and atypical herpesviral encephalitis should be considered as relevant differential diagnosis in iatrogenically immunosuppressed patients in general and especially after high-dose steroid therapy. Moreover, a recent study suggests that atypical presentations of HSV encephalitis might be more frequent than expected so far [26].

In summary, our case represents the reactivation of HSV-1 infection, likely triggered by steroid therapy in the context of a pre-existing severe encephalitis of unknown cause. In order to not overlook newly occurred and potentially treatable entities, continuous re-evaluation of potential differential diagnoses, especially regarding opportunistic infections or reactivation of latent infections, is of utmost importance.

## Data Availability

Data is available upon request. The corresponding author Andreas Osterman should be contacted.

## Abbreviations

ADC: Apparent diffusion coefficient
ADEM: Acute demyelinating encephalomyelitis
AI: Serum CSF antibody specificity index
CNS: Central nervous system
CSF: Cerebrospinal fluid
CT: Computed tomography
DNA: Desoxyribonucleic acid
DOS: Days after onset of symptoms
EEG: Electroencephalography
HSV: Herpes simplex virus
i.v.: Intravenously
ICP: Intracranial pressure
LOD: Technical limit of detection
MRI: Magnetic resonance imaging
NGS: Next-generation sequencing
PCR: Polymerase chain reaction
RNA: Ribonucleic acid
Tid: Ter in die, which in Latin means three times a day
YEL-AND: Yellow fever vaccine-associated neurological disease
